# Thermal Conductivity of Cellulose Fibers in Different Size Scales and Densities

**DOI:** 10.1021/acs.biomac.1c00643

**Published:** 2021-08-17

**Authors:** Mathis Antlauf, Nicolas Boulanger, Linn Berglund, Kristiina Oksman, Ove Andersson

**Affiliations:** † Department of Physics, Umeå University, SE-90187 Umeå, Sweden; ‡ Department of Engineering Sciences and Mathematics, Luleå University of Technology, SE-97187 Luleå, Sweden

## Abstract

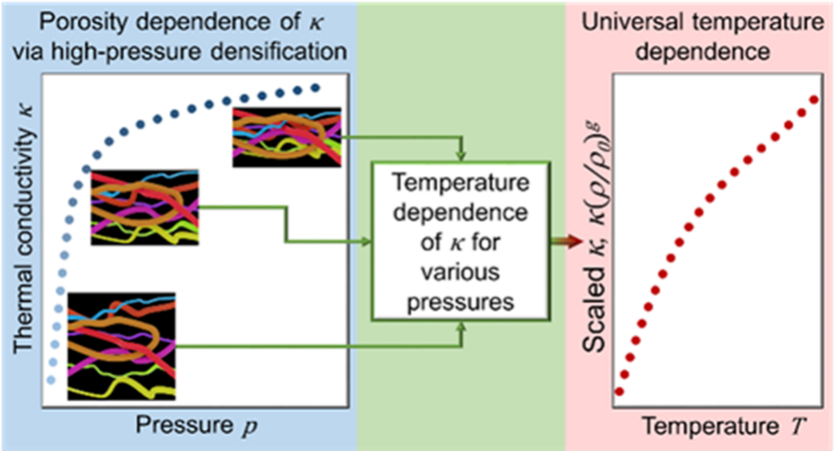

Considering the growing use of cellulose in various applications, knowledge and understanding of its physical properties become increasingly important. Thermal conductivity is a key property, but its variation with porosity and density is unknown, and it is not known if such a variation is affected by fiber size and temperature. Here, we determine the relationships by measurements of the thermal conductivity of cellulose fibers (CFs) and cellulose nanofibers (CNFs) derived from commercial birch pulp as a function of pressure and temperature. The results show that the thermal conductivity varies relatively weakly with density (ρ_sample_ = 1340–1560 kg m^–3^) and that its temperature dependence is independent of density, porosity, and fiber size for temperatures in the range 80–380 K. The universal temperature and density dependencies of the thermal conductivity of a random network of CNFs are described by a third-order polynomial function (SI-units): κ_CNF_ = (0.0787 + 2.73 × 10^–3^·*T* – 7.6749 × 10^–6^·*T*^2^ + 8.4637 × 10^–9^·*T*^3^)·(ρ_sample_/ρ_0_)^2^, where ρ_0_ = 1340 kg m^–3^ and κ_CF_ = 1.065·κ_CNF_. Despite a relatively high degree of crystallinity, both CF and CNF samples show amorphous-like thermal conductivity, that is, it increases with increasing temperature. This appears to be due to the nano-sized elementary fibrils of cellulose, which explains that the thermal conductivity of CNFs and CFs shows identical behavior and differs by only ca. 6%. The nano-sized fibrils effectively limit the phonon mean free path to a few nanometers for heat conduction across fibers, and it is only significantly longer for highly directed heat conduction along fibers. This feature of cellulose makes it easier to apply in applications that require low thermal conductivity combined with high strength; the weak density dependence of the thermal conductivity is a particularly useful property when the material is subjected to high loads. The results for thermal conductivity also suggest that the crystalline structures of cellulose remain stable up to at least 0.7 GPa.

## Introduction

Cellulose is the structural component of the plant cell wall and therefore the perfect choice of polymer for real green high-strength applications and other environmentally benign applications associated with structures based on macromolecules or nanoparticles.^1,2^ In its nano-structured form, nanocellulose, it shows similar properties as other nanomaterials such as nanotubes (e.g., high-strength and large aspect ratio) with the potential of producing cheap, light-weight, strong constructions and/or functional materials while also conforming to the demands of a sustainable society. The continual improvements in the processing of nanocellulose during the last decades have also increased the prospects of new cellulose-based products. Because of its abundance and potential use in a wide range of applications, cellulose and nanocellulose microstructure–property relationships are important, and one key property is the thermal conductivity κ. A basic understanding of its variation with parameters such as density, temperature, and microstructure is vital in heat management applications, and it is also important for modeling heat transfer in applications of cellulose materials.^3−7^ Presently, the variation of κ of cellulose with porosity and density is unknown, or not well quantified, and it is also not known if such a variation is affected by fiber size. Moreover, the temperature variation of κ, and the effect of fiber size, is scarcely studied down to low temperatures; the results will help in understanding the origin of thermal resistivity in cellulose. Here, we solve the issues by using pressure as a variable to determine the effect of porosity and density of κ and to reliably measure the thermal conductivity of cellulose and nanocellulose, as a function of both temperature and pressure. Concurrently, the results show the stability of the crystalline structures of cellulose up to high pressure and high density.

Cellulose resides in the plant cell walls in the form of cellulosic fibers. Each fiber is composed of several microfibrils (5–50 nm in diameter), which, in turn, are composed of elementary fibrils of 3–5 nm in diameter.^1^ The latter are made up of bundles of cellulose chains, and a common model is that these form a repeated pattern of crystalline and amorphous sections along the fibril, but another model with a crystalline core and amorphous shell has also been proposed.^8,9^ In the common model, each crystalline section is of the order of 100 nm in length and it is referred to as a cellulose nanocrystal (CNC). The elementary fibrils consist of up to about 40 individual cellulose molecules/chains, which form a strong network through a hydrogen-bonded network with both intra- and inter-chain bonds.

The hydrogen-bonded network and bond orientations can vary significantly and may give rise to several different structures, or polymorphs, dependent on the source, extraction method, and treatment.^1,2,10^ Native cellulose, cellulose I, is typically divided into two substructures: Iα and Iβ with, respectively, triclinic and monoclinic structures. Iα and Iβ have reported densities of about 1.61 and 1.63 g/cm, respectively. It has been suggested that higher plants consist of various mixtures of these forms with the thermodynamically stable form Iβ being dominant.

Nanocellulose is typically produced by two different main methods: (i) chemical treatment and (ii) mechanical treatment with or without chemical/biological pretreatment.^11^ CNCs can be extracted from cellulose fibers (CFs) through strong acid hydrolysis, which the amorphous parts cannot resist, while the crystalline parts remain stable.^12^ A different form of nanocellulose is produced through mechanical treatment, with or without pretreatments. A common method is to use a high-pressure homogenizer, a microfluidizer, or an ultrafine grinder in which the cellulose pulp is subjected to large shear forces that disintegrate the CFs. This produces cellulose nanofibers (CNFs), or microfibrillated cellulose, which contain both amorphous and crystalline regions and could be up to several micrometers long and 10–100 nm in diameter. CNFs consist of several elementary fibrils, but the number and microstructure can vary, for example, dependent on the processing time.

In this study, we have established the effect of porosity, density, and temperature on κ of nonporous and porous samples of CFs and CNFs derived from a commercial birch pulp by using pressure as a variable.

## Materials and Methods

### Preparation of CFs and CNFs

The starting material was a commercial birch kraft pulp provided by SCA (Munksund, SE) and the nanofiber separation process has been described in detail in a previous study.^13^ The chemical composition was 70 wt % cellulose, 21 wt % hemicellulose, and 5 wt % lignin (see Online Resource 1 of ref (13)). The pulp was diluted to 1.5 wt % and dispersed using a shear mixer Silverson L4RT, (Silverson Machine Ltd., England) before fibrillation. The pulp was processed using an MKCA6-3 Supermasscolloider ultrafine friction grinder (Masuko Sangyo Co., Japan), operated in contact mode with a gap between the two disks gradually adjusted to −90 μm; the processing time was 100 min. The starting pulp fiber dimensions were measured to be 27 ± 7 μm from optical microscopy micrographs. After the fibrillation process, the CNF widths were measured to be 14 ± 6 nm from atomic force microscopy height images.^13^

The CFs and CNFs were dried at 25–35 °C under dynamic vacuum until the weight remained constant (∼4 days). The CNF sample was thereafter ground in liquid nitrogen. After grinding, CNFs were in a mixed form of powder with 1–5 mm sized flakes, whereas the (unground) CF sample was in the form of irregularly shaped granules. Subsequently, the samples were inserted in a press and subjected to ca. 0.1 GPa in a piston-cylinder type die. This produced plates of 39 mm diameter and ca. 4 mm thickness with an atmospheric pressure in-die density of about 1200 kg m^–3^. The samples plates were thereafter stored in a desiccator until characterization by X-ray diffraction, thermogravimetric analysis (TGA), and measurements of thermal conductivity.

### Measurement of Crystallinity with X-ray Diffraction

Accurate determination of the degree of crystallinity of cellulose by means of X-ray powder diffraction (XRD) is challenging and a highly discussed topic to date.^14−17^ The degree of crystallinity of polymer samples is often estimated by X-ray diffraction analysis via the ratio between the crystalline and total (crystalline plus amorphous) areas of the X-ray peaks measured for the solid polymer state but also via slightly modified methods, for example, based on the peak area of the melted (i.e., fully amorphous) polymer.^18,19^ These methods have been carefully reviewed by Kavesh and Schultz,^19^ and because of the limitations of the X-ray methods, they referred the results to apparent degrees of crystallinities. For efficient determination and comparison of the crystallinity of many cellulose samples, Segal et al.^20^ introduced a somewhat different X-ray method and concept—crystallinity index (CI), as “a time-saving empirical measure of relative crystallinity”. (For convenience, we here use CI for all our estimates of the crystallinity.) Because of its simplicity, it has become a widely used method in studies of cellulose. The Segal method is based on the height of the strongest Bragg reflection (*I*_Bragg max_) and the maximum height of the amorphous contribution (*I*_amorph_)
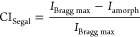
1

For microcrystalline cellulose, the maximum amorphous intensity corresponds to the local minimum at 2θ ≈ 19° between the cellulose Bragg reflections.^16^ Although the Segal method is commonly used, several recent studies suggest that results based on full pattern fitting of the diffraction data provide a better description of the degree of crystallinity.^16,17,21^

XRD data for CF and CNF samples before and after the experiment were collected using Cu Kα radiation (X’Pert3 Powder, PANalytical, Netherlands) in a 2θ range of 5°–60° using a 1/4° fixed diffraction slit and a 2° fixed anti-scattering slit. Samples were prepared on a zero-background silicon sample holder. Instrumental background was measured with identical settings and acquisition time with an empty sample holder as the reference. Full pattern fitting of the instrumental background-corrected XRD data was performed with the WinPLOTR program (Sept-2018, Centre de Diffractometrie X & Institut Laue Langevin, France) of Fullprof Suite (version July-2017).

In the fitting procedure used here, a mathematical background based on a 4th order Chebyshev polynomial function was initially generated. A first approximation of the coefficients was derived from a fit of the experimental patterns with exclusion of the main Bragg peak areas; then, profile fitting (Thomson–Cox–Hastings profile function) for a two-phase mixture of cellulose Iα (*a* = 6.72, *b* = 5.96, *c* = 10.40, α = 118.08, β = 114.80, γ = 80.37) and Iβ (*a* = 7.78, *b* = 8.20, *c* = 10.38, α = 90.00, β = 90.00, γ = 96.55)^22^ was conducted with zero shift (sample displacement) and particle size peak broadening as the only additionally refined parameters. In the last step, background coefficients were refined as well.

**Figure 1:**
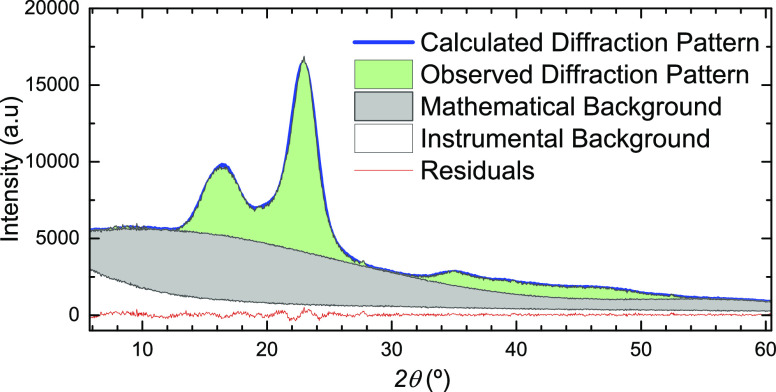
XRD pattern of CNFs before the high-pressure experiment showing the contributions of instrumental background (white), mathematical background = amorphous sample background (gray) and the cellulose Bragg reflections (green). By choosing a 4th order Chebychev polynomial function to model the amorphous sample background (mathematical background), a profile fit of the whole pattern was achieved assuming a mixture of celluloses Iα and Iβ; this resulted in a CI_area_ = 37.4%.

The mathematical background can be treated as the amorphous contribution to the XRD pattern. The ratio of the integrated area below the respective curves, mathematical background (*A*_amorph_) and Bragg reflection contribution (*A*_cryst_), gives CI_area_ (Figure 1)
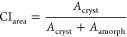
2

Furthermore, we have calculated two different CIs based on the Segal method (eq 1): (i) CI_Segal_ using the instrumental background as a reference for calculating the peak heights, *I*_Bragg max_ and *I*_amorph_, (CI_Segal-inst_) and (ii) CI_Segal_ using the mathematical background as reference (CI_Segal-math_). CI_Area_ as well as CI_Segal-inst_ and CI_Segal-math_ are compiled and compared in Table 1.

**Table 1:** CIs for CF and CNF Samples before and after the High-Pressure Experiment, Which Included Heating up to 423 K at 0.9 GPa (HPHT)^a^

sample	CI_area_ in % (eq 2)	CI_Segal-inst_ in % (eq 1)	CI_Segal-math_ in % (eq 1)
CNF before	37.4	64.8	88.2
CNF after HPHT	31.3	67.5	94.0
CF before	28.0	64.6	94.7
CF after HPHT	31.7	66.0	90.7

a CI_area_ is the result of the above-described full pattern profile fit; it indicates significantly lower crystallinity than the two calculations based on the Segal method. The similar CI values before and after the experiment indicate that no significant changes in crystallinity occurred in the samples during the high-pressure study.

CI_area_ derived by full pattern profile fitting gives ca. 30% lower crystallinity than CI_Segal-inst_, and it differs even more from CI_Segal-math_, which gives crystallinities above 90%. The high apparent crystallinity of CI_Segal-math_ is due to the assumption of no amorphous scattering contribution to the “mathematical background” as opposed to 100% in the calculation of CI_area_. The latter assumption appears to be more realistic; therefore, CI_Segal-math_ probably provides unreasonably high CI, but it corroborates the other two estimates that suggest insignificant CI changes due to the high-pressure treatment. Table 1 shows that CI_Area_ of the CNF sample increased slightly after the high-pressure study, whereas that of CF decreased; CI_Segal-inst_ increased for both samples. The small, and non-systematic, changes suggest that the samples’ microstructures were essentially unaffected by the high-pressure treatment; the best estimate of CI is between 30 and 65%.

### Measurement of Water Content

**Figure 2:**
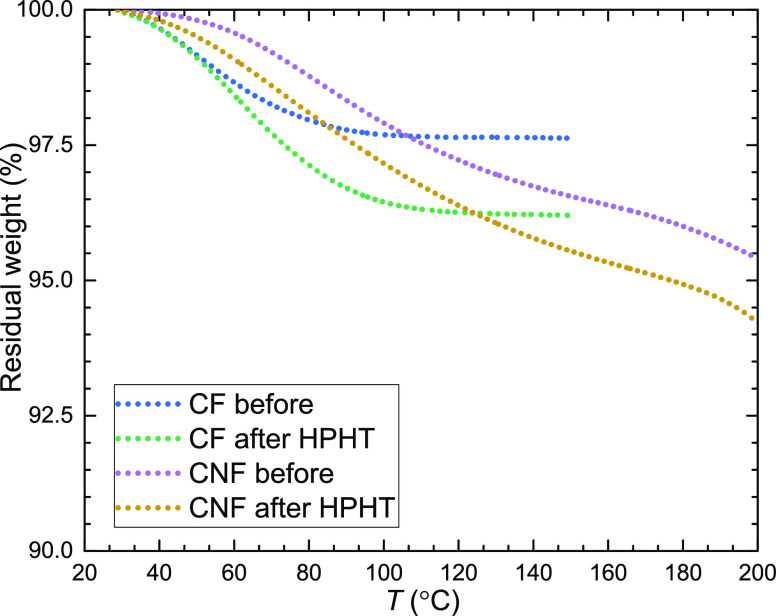
TGA of CFs and CNFs before and after the high-pressure experiment, which included heating up to 423 K at 0.9 GPa (HPHT). Residual moisture in the samples was ca. 3 wt % before the experiment (stored in a desiccator) and up to ca. 5 wt % after the experiment.

TGA (TGA/DSC1 STARe system, Mettler Toledo, Sweden) was used to determine the water content of the CF and CNF samples both before and after the high-pressure experiment (Figure 2). The sample mass was determined in the instrument at a set temperature of 25 °C (sample temperature of about 28 °C). The measurement started with a stabilization step at a set temperature of 25 °C for 20 min followed by a ramp-up to a set temperature of 300 °C for CNFs and 150 °C for CFs at a rate of 1 °C min^–1^.

As shown in Figure 2, CFs release water easier than CNFs, and the TGA data show a constant weight plateau above 373 K. The TGA data of the CNF sample suggest slightly higher water content, but show no clear plateau. The dried CF and CNF samples (about 4 days under dynamic vacuum at temperatures in the range 25–35 °C) contained 2.5 wt % and ca. 3.5 wt % water, respectively. After the subsequent high-pressure study of the thermal conductivity, the water content increased to 4.0 wt % in CFs and ca. 5.0 wt % in CNFs. The origin of this increase is most likely due to exposition to air moisture during sample loading and assembling of the high-pressure equipment and during sample recovery.

### Measurement of Density and Porosity

**Figure 3:**
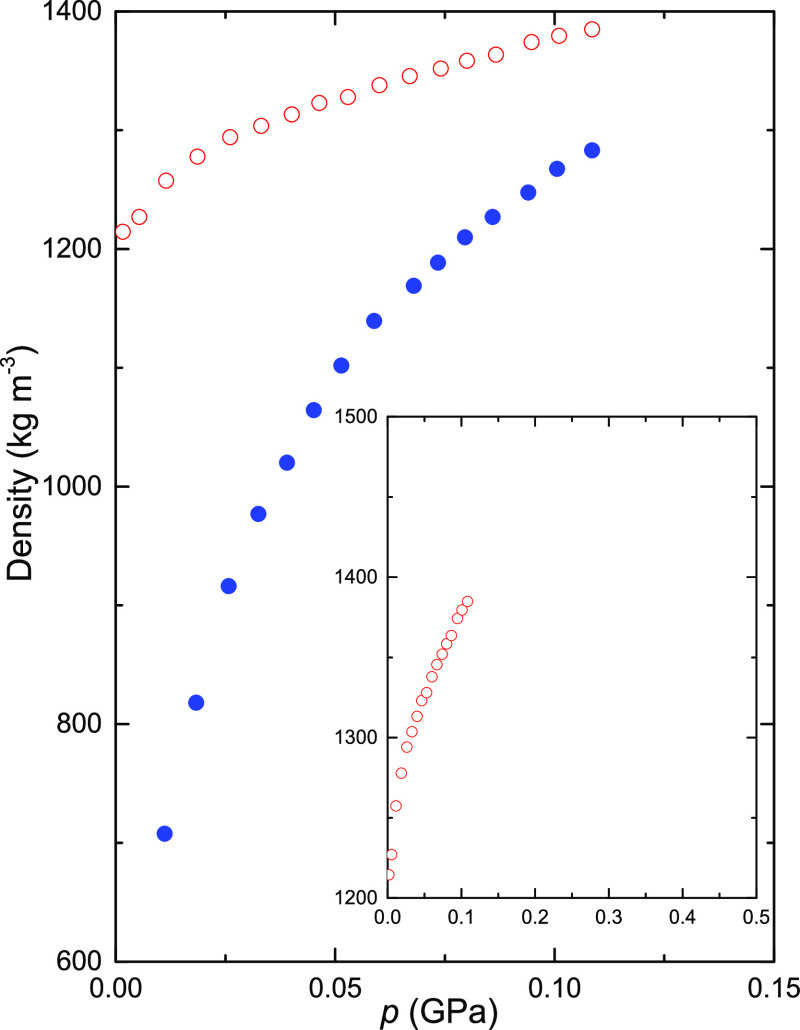
Density of a CF sample plotted against pressure. Blue filled circles show the results during the initial pressurization up to 0.1 GPa. Red open circles show the results during second pressurization—a sample plate lubed slightly with molybdenum sulfide. These measurements mimic the experimental procedure of first producing sample plates by pressurization up to 0.1 GPa and thereafter repressurizing the plates while measuring the thermal conductivity. The same results shown in the inset suggest that compressed CF particles attain the nonporous density of slightly higher than 1500 kg m^–3^ at a pressure below 0.5 GPa, which is also indicated by the measurements of thermal conductivity.

The sample density and porosity were determined in a separate high-pressure experiment. A piston cylinder device of 39 mm internal diameter was mounted in a small hydraulic press together with a displacement sensor and a load cell. The sample was first pressure-cycled up to ca. 0.1 GPa, lubed, and thereafter again pressurized (Figure 3) to mimic the preparation of the sample plates and treatment of the samples during the measurements of the thermal conductivity. The sample porosity ε, or void content, was calculated from
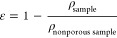
3where ρ_nonporous sample_ is the density of the sample without voids or the nonporous density, which is about 1500 kg m^–3^ at atmospheric pressure and 1510 kg m^–3^ at 0.06 GPa. The measurements provide data for sample density as a function of pressure and, thus, porosity as a function of pressure up to 0.1 GPa. The measurements of a CF sample gave a porosity of 0.11 and density of 1340 kg m^–3^ at 0.06 GPa. We use the same data for CNF as for CF to calculate the density dependence of the thermal conductivity. A calculation of the compressibility of the samples in the pressure range above 0.05 GPa (without lube) gave the same compressibility for CNF and CF samples to within 1.5%, but with a standard error of 7%. (We estimate that the inaccuracy in the compressibility could be 20%, which gives the same inaccuracy in the density dependence of the thermal conductivity. The true densities of CNF and CF are likely the same.)^23^

### Measurement of Thermal Conductivity

The transient hot-wire method was used to measure the thermal conductivity κ with an estimated inaccuracy of ±2%.^24,25^ The hot-wire probe was a 0.1 mm-diameter Ni wire, which was inserted in a custom-made, ca. 13 mm deep and 39 mm internal diameter, Teflon sample cell. A wire of ca. 40 mm length was sandwiched between two pre-pressed plates of CFs or CNFs (see above for sample preparation) and sealed with a tightly fitting Teflon lid. The cell was mounted on a bottom piston and inserted in a pressure cylinder of 45 mm internal diameter. The whole assembly was thereafter transferred to a fully automatic hydraulic press, which supplied the load. Temperature was varied by cooling or warming the whole pressure vessel using liquid nitrogen and an external electric heater; it was measured using an internal chromel versus alumel thermocouple. Pressure was determined from the load/area with an empirical correction for friction which has been established using the pressure dependence of the resistance of a manganin wire. The inaccuracies in temperature and pressure are estimated as 0.5 K and 40 MPa (at 1 GPa), respectively.

In each measurement of κ, the Ni-wire (hot-wire) was subjected to a 1.4 s duration heat-pulse of nominally constant power, and its electrical resistance was measured as a function of time. Subsequently, the temperature rise of the wire was calculated by using the relation between its resistance and temperature; the wire acted as both a heater and a sensor for the temperature rise. The analytical solution for the temperature rise with time was fitted to 29 measured data points for the hot-wire temperature rise with κ and the heat capacity per unit volume as fitting parameters. The heat capacity per unit volume is determined (fitted) mainly by the first of the 29 measurement points,^24^ and for two CF samples it differed by 30% when the thermal conductivity differed only by 3%. Because of the observed discrepancy in the heat capacity per unit volume, we used it only as a fitting parameter and do not report the data. Measurements of κ of CFs and CNFs were done on the same batch of pulp. However, we have also compared results of CFs of two different batches and κ of these differed less than 5%. In the measurements of κ, the heat wave penetration depth is 1–2 mm in the radial direction of the 40 mm long hot-wire probe, which is immersed in the sample. Consequently, we here provide κ of a sample with randomly oriented microfibers. (Since the heat pulse travels in the radial direction of the hot-wire, it travels both along and perpendicular to the direction of the applied pressure; therefore, compression-induced orientation ordering of fibrils, if any, will not significantly affect the results.)

## Results and Discussion

CF and CNF samples were initially pressurized from atmospheric pressure to 0.06 GPa at room temperature. Subsequently, the samples were temperature-cycled between room temperature and low temperatures at 0.06 GPa to determine the temperature dependence of the thermal conductivity κ of porous samples, that is, samples which were not fully compacted. The sample porosity ε, or void content for CFs at 0.06 GPa, was calculated from eq 3 (see Materials and Methods), which gave a porosity of ε = 0.11 and a density of 1340 kg m^–3^; this should be a good estimate also for the CNF sample. All κ results reported here relate to CF and CNF samples with randomly oriented microfibers (see Materials and Methods).

**Figure 4:**
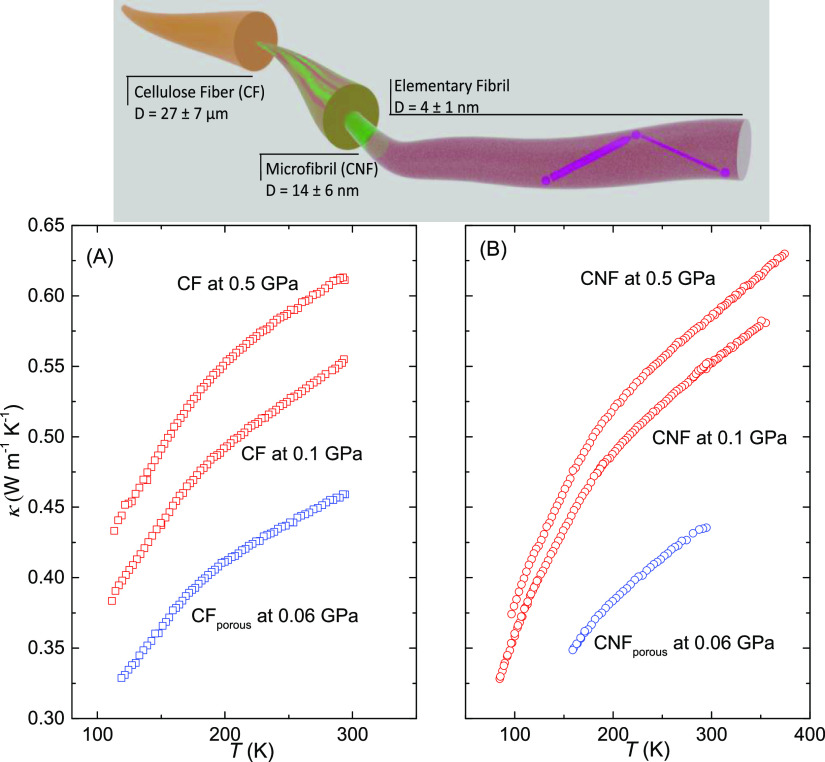
Thermal conductivity plotted against temperature at the pressures indicated: (A) CF and (B) CNF. The porous samples have an estimated porosity ε = 0.11 and a density of 1340 kg m^–3^. The top panel shows a schematic view of the frequent phonon scattering due to the boundaries and amorphous fractions of the nano-sized elemental fibrils (magenta dots), which causes an amorphous-like (positive) temperature dependence of κ.

Figure 4 shows the results for κ of the CF and CNF samples at 0.06 GPa. The temperature dependencies of κ are identical, but CF shows an ca. 6% larger magnitude. These results with low and constant, or slightly decreasing, κ(*T*) on cooling are typical of structurally disordered materials such as amorphous (e.g., glasses) and semicrystalline materials with low degree of crystallinity. κ(*T*) of amorphous states is roughly described by a function κ ∝ *T*^–*x*^, with *x* being slightly negative or close to zero. This is much different from the behavior of single crystals and polycrystalline materials, which typically show κ ∝ *T*^–1^ at similar temperatures. As a rough distinction, one may therefore refer to κ(*T*), which is described by a function with *x* close to zero or negative as amorphous-like (or glass-like) and κ(*T*) described by positive *x* as crystal-like. The reason for the different behaviors is that κ of crystals is limited by phonon–phonon (Umklapp) scattering, and the number of phonons increases with temperature,^26^ whereas phonon propagation in amorphous materials is limited by (temperature-independent) structural disorder (see calculations and Figure 3 in ref (27)).

Amorphous and semicrystalline polymers with low crystallinity show amorphous-like κ, whereas polymers with a high degree of crystallinity are expected to show crystal-like κ.^18^ However, a general description of the change from amorphous-like to crystal-like κ(*T*) in terms of CI is not possible. Amorphous-like κ has been found for several semicrystalline polymers such as nylon-6,^28^ polyethylene (PE),^29^ and poly-l-lactide.^30^ In all these cases, it is possible to significantly increase CI by thermal treatments to study the effect of crystallinity on κ. For example, κ(*T*) of nylon-6 changes from amorphous-like to crystal-like behavior when CI increases from 30 to 56%. However, in a study of poly-l-lactide with CIs in the range 0–56%, κ(*T*) was amorphous-like in the entire range.^30^ Such different behaviors may be due to differences in the microstructures. More specifically, in a study of PE, it was shown that the crystal-like behavior of κ was strongly promoted by an increase of the nano-sized lamellar thickness.^29^ Two different PE samples with similar CI, but one with larger lamellar thickness, displayed significantly different degrees of crystal-like behavior of κ(*T*) (*x* = 0.71 and 0.35, respectively). Thus, even if CF and CNF samples may show high CI, which here is in the range 30–65% (see Materials and Methods), κ(*T*) may still be amorphous-like due to the inherent nano-sized structure of CFs. That is, their building blocks of thin elementary fibrils (3–5 nm in diameter) with crystalline regions that are either separated by amorphous regions ∼100–900 nm apart along the microfibrils^1^ or have less-ordered (amorphous) surface regions of cellulose chains.^8,9^ Indeed, Adachi et al.^31^ have recently shown that κ(*T*) of individual CNFs is amorphous-like and suggested that it is due to their nano-sized structure. Individual CNFs show κ = (2.2 ± 1.2) W m^–1^ K^–1^ at 300 K and a remarkable (weak) decrease on cooling,^31^ which is corroborated by molecular dynamics simulations.^32^ We can conclude that the amorphous-like κ(*T*) of the CF and CNF samples are in qualitative agreement with Adachi et al.’s^31^ result for κ along individual CNFs; the amorphous-like κ(*T*) is therefore likely associated with the inherent nano-sized structure of CFs and in origin similar to that of other nanomaterials such as carbon nanotubes^33^ and Si nanowires.^34^ (The relative decrease of κ for the CF and CNF samples of ca. 30% down to 100 K is in rough quantitative agreement with the corresponding result of κ along individual CNFs.^31^) This also explains the identical temperature behavior and small difference in magnitude of κ(*T*) for CFs and CNFs. Fibrillation should increase the thermal resistance in the sample, but since κ is strongly limited by the inherent nano-sized structure and the already existing microfibril boundaries, it does not affect the temperature dependence of κ and only weakly its magnitude (ca. 6%). Thus, boundaries of fibrils appear as a possible source for strong, temperature-independent, phonon scattering; in this case, the phonon mean free path becomes limited to the distance between boundaries. Structural studies have also shown increasing disorder in chain packing and hydrogen bonding outward from the center of elementary fibrils,^35^ which further decreases the crystalline size in this direction. Figure 4 shows a schematic view of phonon-boundary scattering due to the nano-size of elementary fibrils, where the phonon mean free path can only be significantly longer than the diameter of the fibrils for propagation along the fibrils. (We note that the dominant phonon wavelength, λ_dominant_ ≈ *hvk*^–1^*T*^–1^ where *v* is the phonon velocity, *T* is the temperature, *k* is Boltzmann’s constant, and *h* is Planck’s constant, is of the order of the diameter of an elementary fibril, which makes the phonon concept questionable for phonon propagation across a fibril.)

In order to significantly reduce voids and form a dense random network of CNFs and CFs, the samples were thereafter pressurized to 0.5 GPa; this produced nonporous, or close to nonporous, samples, which were temperature-cycled at 0.5 GPa (Figure 4). Our conclusion of a nonporous sample at 0.5 GPa is supported by our measurements of density versus pressure, which suggest that compressed CFs reach the estimated nonporous density of ca. 1500 kg m^–3^ at a pressure below 0.5 GPa (Figure 3). Moreover, measurements of a sample of microcrystalline cellulose by Sun^36^ suggest that it achieved the nonporous density after applying a compaction pressure slightly above 0.1 GPa. As depicted in Figure 4, nonporous CF and CNF samples with an estimated density of 1560 kg m^–3^ at 0.5 GPa and porous CF and CNF samples (1340 kg m^–3^) show identical temperature behavior of κ. To estimate the density of nonporous CFs and CNFs, we used the bulk modulus of cellulose *B* = 11.6 GPa in the 0.2–0.6 GPa range^37^ and an estimated atmospheric nonporous density of 1500 kg m^–3^.

Finally, the samples were pressurized up to 0.9 GPa, depressurized to near ambient pressure, and repressurized up to 0.1 GPa at room temperature. This produces a well-compacted sample with an estimated density of about 1510 kg m^–3^ at 0.1 GPa. The results on temperature cycling nonporous CNFs at 0.1 GPa are also depicted in Figure 4B. As shown, the temperature behavior is the same for porous CNFs at 0.06 GPa and nonporous CNFs at 0.5 GPa, but the magnitudes of κ differ due to the different densities. Results for similarly treated CFs are shown in Figure 4A; the behavior is the same as that for CNFs. However, the results at 0.1 GPa are lower than expected for a nonporous sample, that is, the results suggest that the CF sample was not in nonporous form at 0.1 GPa. In this case, the sample was not heated above room temperature at high pressure before the temperature cycle at 0.1 GPa. This suggests that slight heating at high pressure is required to keep the near nonporous form at lower pressures; after heating to 373 K at 0.5 GPa, the subsequently measured κ of CFs increased by about 3.5% at 0.1 GPa.

**Figure 5:**
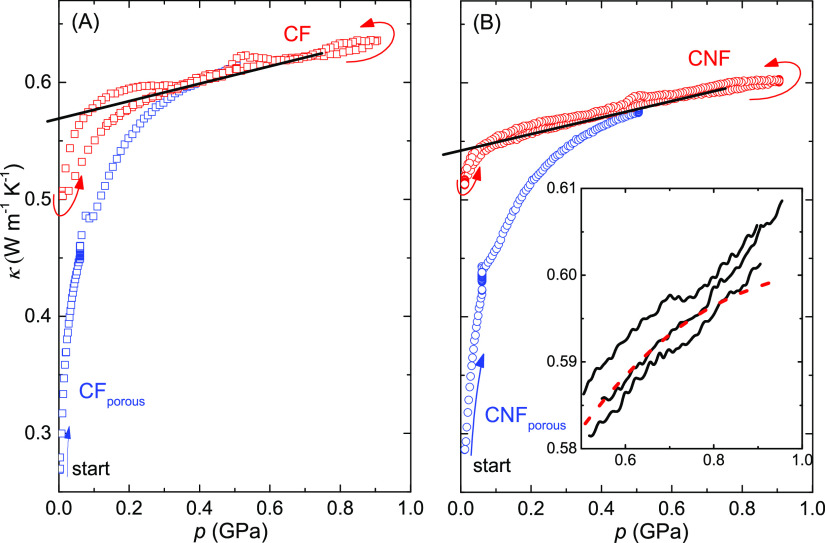
Thermal conductivity plotted against pressure at 295 K: (A) CF and (B) CNF. The increase in κ of porous samples at 0.06 GPa is due to a sluggish relaxation (densification) observed during 14 h (CF) and 10 h (CNF) measurements at constant temperature and pressure. (The small bump in κ observed at 0.5 GPa on depressurization is due to an exothermic transition in the sample cell material—Teflon; this causes a slight rise in Teflon temperature and volume.) The black lines show extrapolations of κ(*p*) of nonporous samples down to atmospheric pressure, which yields 0.57 W m^–1^ K^–1^ for CF and 0.54 W m^–1^ K^–1^ for CNF. (κ of nonporous samples varies typically linearly with pressure in a pressure range with constant compressibility.^28,29^) The inset shows an expanded view of results for three separate runs of CNF measured on increasing pressure (solid lines) and a dashed red line representing the typical pressure dependence of κ in the absence of a transformation in the sample.

To determine the effect of density and porosity, we use data on pressure cycling at room temperature. Figure 5 shows the results on pressurization of CF and CNF samples up to 0.9 GPa, with intermediate temperature cycles at 0.06 and 0.5 GPa (Figure 4). The results show that κ of the CF sample is only about 6% higher than that of the CNF sample. The strong initial increase of κ is mainly due to the elimination of voids, but with some contribution from improved thermal contact between the sample and probe for pressures below about 0.05 GPa. The latter is indicated by a decreased error in the fits of the temperature rise of the probe (see Materials and Methods). When the increase of pressure halted at 0.06 GPa, κ of the CF and CNF samples slowly increased due to sluggish sample densification; κ of CNFs increased by 4% and CFs slightly less during a period of about 10 h. A fit of a single exponential function gave a relaxation time of 3.6 and 2.9 h for CNFs and CFs, respectively. At the second pressurization, after decreasing to near ambient pressure from 0.9 GPa, the samples were in near nonporous form, and for pressures above about 0.5 GPa, κ retraced the data measured at the initial pressurization; this indicates that both samples were indeed well-compacted at 0.5 GPa during the first pressurization. However, in the pressure range 0.15–0.5 GPa, κ(*p*) of CFs and CNFs differ during the initial pressurization. The approach to reach the nonporous state is somewhat more gradual for CNFs than for CFs. The CF sample reached the (near) nonporous state at about 0.35 GPa, whereas the CNF sample approached the state at 0.5 GPa. We attribute this to differences in particle size/shape and the mixture of flakes and powder in CNF (see the Materials and Methods section). Still, in the nonporous and porous states, CF and (defibrillated) CNF show essentially equal thermal conductivity with identical temperature dependence and similar pressure dependence, which seems to suggest good interfacial contact between the fibrils/fibers in the range studied here.

Strong densification of materials can induce structural changes, and such are typically observed as discontinuous changes in κ. A continuous change of κ with no significant abrupt changes in either the temperature dependence (Figure 4) or the pressure dependence (Figure 5) suggests that the crystal structure of the material does not change. A more detailed plot of the results measured on isothermal pressurization does show a weak indication of an increased pressure dependence of κ. This is an atypical behavior, which was reproduced in several pressure runs of CNFs (see inset in Figure 5B) and CFs. Because of the decreasing compressibility of materials at high pressure, the increase of κ(*p*) due to densification typically levels off. The accelerated increase of κ(*p*) near 0.75 GPa may therefore be due to a gradual, or second-order, transformation, but the change is too small for conclusive evidence of a transformation. Although this type of behavior has not been noted under similar conditions in other polymer materials,^28,29^ it occurs near a transition in Teflon, making the interpretation of the finding further uncertain.

Besides possible structural changes, high-pressure treatment of materials may induce both an increased degree of crystallinity and a changed orientation of crystals as, for example, seen in nylon-6 treated at 1 GPa and 500 K,^28^ but such changes are less likely to occur in polymers kept at temperatures near room temperature and below. XRD patterns measured before and after the high-pressure studies suggest no change in CI (Table 1) or crystal structure of the CF and CNF samples despite heating up to 423 K at 0.9 GPa to explore the possibility of such changes (see details below). Consequently, the results presented in Figures 4 and 5 pertain to porous and (near) nonporous CF and CNF samples with the as-produced degree of crystallinity. This inference is supported by the repeatability of the results after forming the nonporous state.

To compare with literature results, we use data for nanocellulose, which have been studied recently. We reiterate that the results measured in this study relate to random networks of CNFs and CFs, which is different from the structure of sheets/papers in which fibers typically show a preferred orientation. The effect of both changing crystal size and direction of heat flow on κ of (non-woven) nanocellulose sheets/papers made from various sources such as tunicate, bacterial cellulose, cotton, and wood pulp was studied and reviewed by Uetani and co-workers;^3,38^ through-plane values of κ are in the range 0.3–0.5 W m^–1^ K^–1^, independent of the source material, and source-dependent in-plane values are in the range 0.6–2.5 W m^–1^ K^–1^. Thus, κ of our nonporous samples of ca. 0.57 W m^–1^ K^–1^ for CF and 0.54 W m^–1^ K^–1^ for CNF (Figure 5) is in between that through-plane and that in-plane, whereas κ of porous CF and CNF samples is in the range of that through-plane of a nanocellulose sheet; it is also similar to values reported by Diaz et al.^4^ for CNC films: 0.22–0.53 W m^–1^ K^–1^. The low value for κ of CNC films (0.22 W m^–1^ K^–1^) was reported for a film formed by self-organization by slowly evaporating a diluted aqueous CNC suspension under ambient conditions. Films formed by casting CNC suspensions under different shear rates showed increasingly higher values with increasing shear rate, which was attributed to shear-induced ordering.^4^ Diaz et al.^4^ also studied κ of a single CNC (Iβ) by molecular dynamics simulations and reported κ = (5.7 ± 0.9) W m^–1^ K^–1^ along the fiber direction and κ = (0.72 ± 0.12) W m^–1^ K^–1^ in the traverse direction. As mentioned above, Adachi et al.^31^ reported κ = (2.2 ± 1.2) W m^–1^ K^–1^ along individual CNFs at 300 K, which is consistent with our result (0.54 W m^–1^ K^–1^) for a dense network of randomly oriented CNFs. A model of such network with negligible thermal resistance between the fibers suggests κ = κ_0_/π,^39^ where κ_0_ is the thermal conductivity along the fiber, that is, κ = (0.7 ± 0.4) W m^–1^ K^–1^ for a dense CNF network based on Adachi et al.’s result.^31^ Conversely, our results combined with the model^39^ suggest κ = 1.7 W m^–1^ K^–1^ along individual CNFs.

To quantify the effect of density and porosity on κ, we use the Bridgman parameter *g*, which is defined by
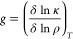
4where ρ is the density.

**Figure 6:**
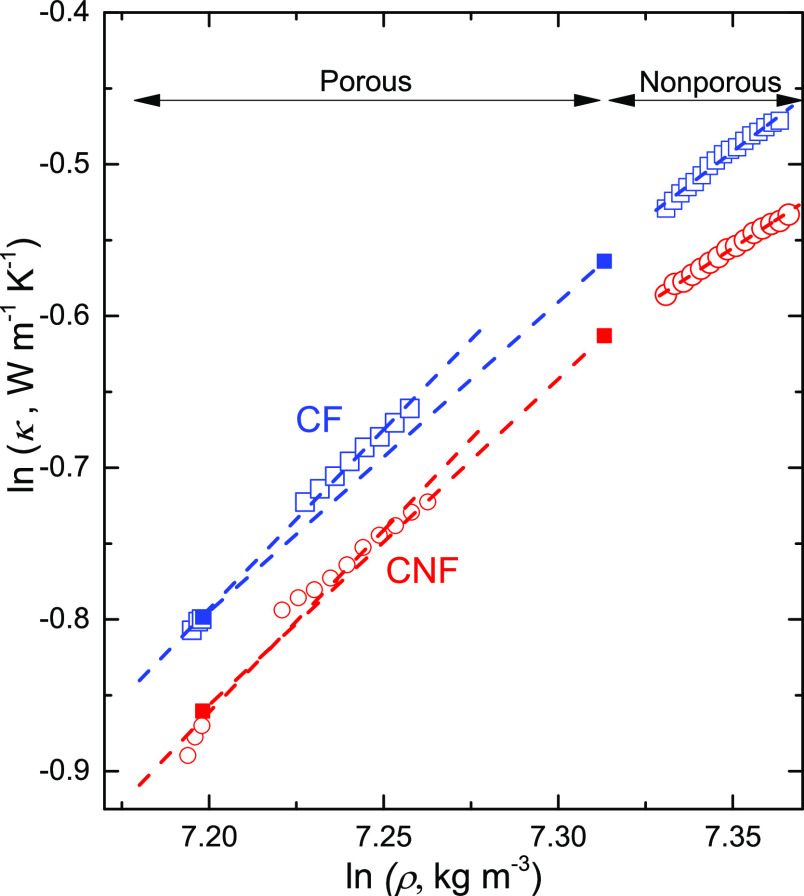
Natural logarithm of the thermal conductivity plotted against the natural logarithm of density. Results for porous and nonporous CF (squares) and CNF (circles) samples. The dashed lines represent linear fits with the Bridgman parameter *g* corresponding to the slope. The filled symbols represent measured data at 0.06 GPa and values for the nonporous samples at 1 atm with an estimated density of 1500 kg m^–3^. To calculate the density of nonporous CFs and CNFs, we used the bulk modulus of cellulose *B* = 11.6 GPa in the 0.2–0.6 GPa range.^37^

To determine the Bridgman parameter of nonporous CF and CNF samples, we used the data on pressurization in the 0.2–0.6 GPa range combined with density values measured for cellulose (cotton).^37^ In this case, κ was measured after the samples had been subjected to 0.9 GPa at room temperature and 373 K at 0.5 GPa, which should ensure that the samples were nonporous. The results are presented in Figure 6. The CF and CNF samples show similar density dependence *g* = 1.8 and 1.5, respectively, which is in the lower range of the density dependences measured for polymers, especially considering that these are semicrystalline states. Typically, amorphous, liquid, and glassy polymers show values near *g* = 3,^40,41^ whereas semicrystalline polymers show somewhat higher values, for example, low-density PE with *g* ≈ 5 and high density PE with *g* ≈ 10 at 100 °C,^41^ with few exceptions such as semicrystalline isotactic poly(propylene) with *g* = 1.85.^42^ Our direct measurements of *g* in the porous states (ε < 0.11) support the finding of *g*-values near 2 for CF and CNF samples.

For the porous CF and CNF samples, we used the data for κ on the initial pressurization; we excluded data at the lowest pressure, which are subjected to gradually improved thermal contact between the probe and sample as well as initial data after temperature cycling, which are subjected to the change of friction as the piston movement changes direction. The data, which are shown in Figure 6, give a value for *g* of 2.4 for both CNFs and CFs. Moreover, we can calculate an average value using data at low pressure (0.06 GPa) and the results for the nonporous samples at atmospheric pressure (estimated density = 1500 kg m^–3^), which gives *g* = 2.0 for CF and *g* = 2.2 for CNF (Figure 6). These results suggest that porous CFs and CNFs both have unusually low *g*-values.

From eq 4, it follows that
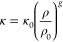
5where we find *g* = 2.0–2.4 in the porous range for both CFs and CNFs and values slightly below 2 in the nonporous states. Considering the similar pressure and temperature behavior of κ for CFs and CNFs and their close structural relationship, the best approach for determining the most accurate density dependence seems to be to use an average value, which gives *g* = 2.0 ± 0.5. Thus, the combined data suggest that κ of CNF and CF samples increases 2% for every percent increase of density. We note that the unusually weak density dependence for a partly crystalline material may possibly also be a consequence of the inherent nano-sized structure of cellulose. This feature of cellulose, combined with its strength, makes cellulose particularly useful as a thermally isolating material in applications involving high loads.

**Figure 7:**
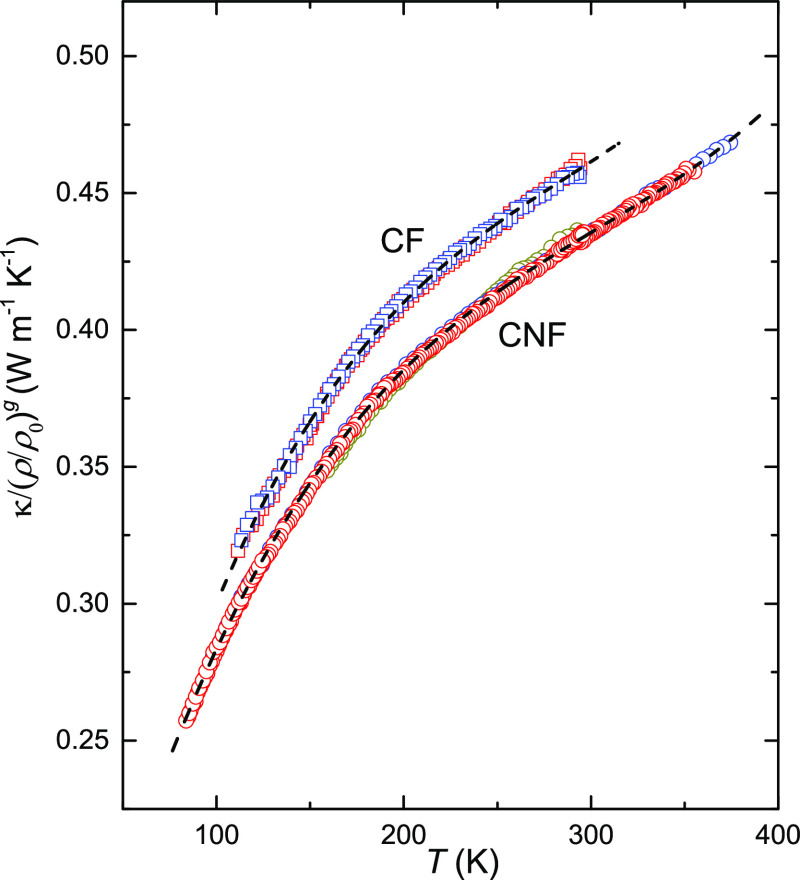
Density-scaled thermal conductivity, κ/(ρ/ρ_0_)^*g*^, plotted against temperature, see eq 5: (circles) data for CNFs measured at 0.1 GPa (ρ = 1514 kg m^–3^) and 0.5 GPa (ρ = 1566 kg m^–3^) were scaled using *g* = 1.95 and *g* = 1.90, respectively; the data collapse on the data for porous CNFs, ρ_0_ = 1340 kg m^–3^, measured on cooling at 0.06 GPa; (squares) corresponding data for CFs with *g* = 1.88 and *g* = 1.55, respectively. The dashed lines represent third-order polynomial fits to all data sets for CNFs and CFs, respectively.

Equation 5 can be used for density scaling of isobaric data; Figure 7 shows the results of the densified samples scaled to the density of porous CF and CNF samples at 0.06 GPa. The good agreement shows that the temperature behavior is unaffected by porosity and density. These density-scaled data are well described by a third-order polynomial. The general expression for κ of porous and nonporous CNFs as a function of temperature and density in the range 1340–1560 kg m^–3^ is given by
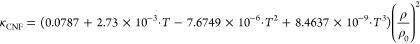
6with the reference density ρ_0_ = 1340 kg m^–3^ and the temperature *T* in Kelvin. A best fit of the same function to the data of CFs in a narrower temperature range gives different coefficients but the temperature behavior is similar to that of CNF. To simplify the description, we can therefore rescale eq 6 using a constant, κ_CF_(*T*) = *C*κ_CNF_(*T*), where *C* = 1.065, which describes the best third-order polynomial fit of κ_CF_(*T*) to within 0.5% in the 100–300 K range.

Since high-pressure high-temperature(HPHT) treatments of polymers such as nylon-6^28^ and PE^29^ have produced highly crystallized states and significant changes in the microstructures (e.g., increased lamellar sizes) as well as new crystalline structures, we have also investigated the possibility of similar changes for CNFs; the prospects of such transformations are best at high temperatures and high pressures. The CNF sample was therefore heated up to 423 K at 0.9 GPa, which is close to the maximum capacity of the pressure vessel. The temperature was chosen as the highest possible for long-time annealing without the risk of rapid decomposition processes. An increase in crystallinity and/or crystal–crystal transformations, under highly densified conditions, will most likely cause increasing values for κ. However, the measurements showed weakly decreasing κ with time, suggesting a slow decomposition process, and the treatment was therefore aborted; the sample was cooled down to room temperature and recovered under ambient conditions for characterization by XRD and TGA. The XRD of the recovered sample showed no significant changes in the pattern compared to that before the high-pressure experiment (see Materials and Methods). Consequently, during the study here, we find no indications of significant irreversible changes in the microstructure up to 0.9 GPa, but a weak increase in κ near 0.75 GPa on pressurization at room temperature may possibly be due to a reversible phase transition.

## Conclusions

The thermal conductivities of CF and CNF samples show positive temperature dependence or amorphous-like behavior. We attribute this to the nano-sized building blocks, elementary fibrils, of the fibers and the amorphous-like κ of individual nanofibers (or microfibrils). The elementary fibrils limit the phonon mean free path to a few nanometers for heat conduction across fibers, and it can only be significantly longer for highly directed heat conduction along the fibers. This explains the identical temperature dependence of κ of CF and CNF samples and accounts for the size of κ for a random dense network of CNFs and CFs, that is, κ is virtually independent of the fiber size. At 295 K, κ is ∼0.54 W m^–1^ K^–1^ for a dense random network of CNFs (ρ = 1500 kg m^–3^) and ∼0.43 W m^–1^ K^–1^ for a porous network (ρ = 1340 kg m^–3^, porosity ε = 0.11); values for the corresponding networks of CFs are about 6% higher. The result for a dense random network of CNFs is in good agreement with the corresponding result based on κ along individual CNFs.^31^

The temperature behavior of κ of CF and CNF samples is independent of density and porosity in the range 1340–1560 kg m^–3^; the universal temperature and density dependencies for CNF are well described by the relation κ_CNF_ = (0.0787 + 2.73 × 10^–3^·*T* – 7.6749 × 10^–6^·*T*^2^ + 8.4637 × 10^–9^·*T*^3^)·(ρ/ρ_0_)^2^, where ρ is the sample density and ρ_0_ = 1340 kg m^–3^ is a reference density; κ of CF is described by the same function but is a factor of 1.065 larger. Both CFs and CNFs show an unusually weak density (pressure) dependence of κ. It varies as the density squared, that is, κ increases by 2 for a 1% increase in density, whereas it typically varies proportionally to the cube of density for amorphous polymers and even stronger for semicrystalline polymers. This property and the high thermal resistivity across fibers, combined with its high strength, makes cellulose particularly interesting in applications involving high loads.

The lack of significant discontinuous changes in κ and its derivative during pressure and temperature cycling of CF and CNF samples suggests that the crystalline structures of cellulose are stable up to at least 0.7 GPa and likely up to 0.9 GPa. Moreover, the essentially unchanged X-ray pattern of the recovered CF and CNF samples shows that no significant irreversible changes occurred during treatment at 0.9 GPa for temperatures up to 423 K. In particular, the degree of crystallinity of cellulose remained unchanged by this treatment.
